# Critical Behaviors for Perioperative Improvement Teams

**DOI:** 10.1097/AS9.0000000000000300

**Published:** 2023-06-28

**Authors:** Christina T. Yuan, Tasnuva M. Liu, Benjamin Eidman, Della M. Lin, Elizabeth C. Wick, Michael A. Rosen

**Affiliations:** From the * Armstrong Institute for Patient Safety and Quality, Johns Hopkins University School of Medicine, Baltimore, MD; † Department of Health Policy and Management, Johns Hopkins Bloomberg School of Public Health, Baltimore, MD; ‡ Department of Surgery, John A. Burns School of Medicine, Honolulu, HI; § Department of Surgery, University of California, San Francisco, San Francisco, CA; ‖ Anesthesiology and Critical Care Medicine, Johns Hopkins University School of Medicine, Baltimore, MD.

## Abstract

Effectively leading perioperative safety and quality improvement requires a multidisciplinary team approach. However, leaders are often left without clear guidance on how to assemble and manage teams in these settings. We employ a Delphi process to prioritize specific behavioral strategies surgical safety and quality leaders can use to improve their chances of success implementing improvement efforts. We present the panel’s consensus practical guidance on designing, managing, sustaining, training their teams as well as managing team boundaries and the organizational context.

## INTRODUCTION

When implementing perioperative improvement projects like enhanced recovery pathways, a common recommendation is to assemble a multidisciplinary team to lead the effort. Involving key stakeholders, building shared visions, and establishing a collaborative environment can increase uptake of interventions.^1^ However, it is not enough to acknowledge the need for a multidisciplinary team; leaders must know how to effectively design and manage these teams. Literature on team performance suggests many factors contribute to high-functioning teams, such as mindfully composing teams, effectively managing relationships, and promoting team learning.^2^ What remains unclear is what behaviors should be prioritized and how to enact them in perioperative services. The existing science of teams and leadership is vast, and the guidance it provides can be ambiguous. These represent significant challenges to the translation of this science to perioperative improvement. We sought to generate parsimonious, practical, consensus-based guidance on how to manage perioperative safety improvement teams.

## METHODS

We conducted a Delphi study between October 2018 and March 2019, with a 14-member panel of recognized experts in research, management, or perioperative improvement. Participants were selected to represent a range of professional roles (surgeons, anesthesiologists, nurses, health care administrators, and researchers) and hospital types, which varied by teaching status, size, and geographic location.

**FIGURE 1.**
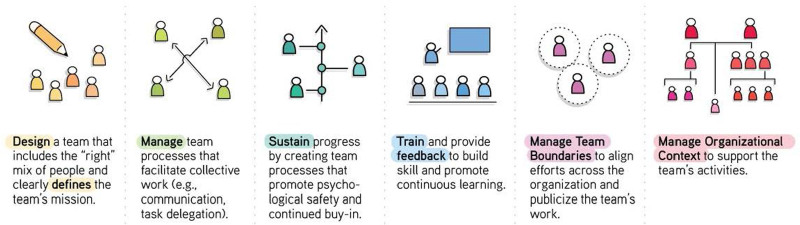
Six critical teamwork domains for perioperative improvement teams.

The Delphi method is a systematic, in-depth qualitative methodology to elicit expert opinions and generate consensus on a topic.^3^ Three rounds of surveys were administered using Qualtrics. In Round 1, panelists ranked the importance of specific factors from the team performance literature, including team leadership structures and processes,^4^ characteristics of externally oriented teams (X-teams),^5^ the Team Diagnostic Survey,^6^ and the Organizational Readiness to Change Assessment.^7^ Panelists were also given the opportunity to suggest additional factors. The 63 factors for Round 1 were organized into six critical domains: (1) design and define,^2^ manage,^3^ sustain,^4^ training and feedback,^5^ manage boundaries, and^6^ manage organizational context (Fig. 1). For each domain, panelists selected what they believed to be the top 5 factors for high-performing teams. We defined consensus as >50% agreement among panelists. In Round 2, panelists received the list of top-rated factors from Round 1 and were asked to “provide practical guidance on how leaders can support these critical drivers of high-performing teams.” Practical guidance was defined as tips, tactics, or approaches based on the panelist’s first-hand experience in improvement efforts. In Round 3, participants were asked to rate the practical guidance provided in Round 2 as either critical (“Must Do”), facilitating (“Usually important, but not always”), or non-critical (“Nice to do but not essential”).

### Analysis

Four (CY, TL, BE, MR) study team members with expertise in patient safety, quality, and team science synthesized the qualitative responses from Round 2, grouping overlapping recommendations. A list of 50 “leader behaviors” and practical tips were generated for Round 3. All 14 panelists participated in all 3 rounds.

## RESULTS

### Round 1: Rating Factors From the Team Performance Literature

Delphi panelists reached consensus on 25 critical drivers of high-performing teams. In the “Define and Design” domain, the highest ranked factors were to articulate a clear team purpose (86% consensus), select the “right mix” of skills (86%), and include highly motivated team members (86%). Other highly ranked factors included project leaders setting challenging but realistic goals (79% consensus; “Manage” domain), representing the team when engaging other parts of the organization (86% consensus; “Manage Boundaries” domain), and seeking different perspectives when solving problems (79% consensus; “Sustain” domain).

### Round 2: Generating Examples of Leader Behaviors

This section provides some of the leader behaviors with supporting quotes from panelists organized by the 6 critical teamwork domains.

#### Domain 1: Design and Define

One leader behavior was “involving key stakeholders and roles.” One panelist provided the following practical guidance: “We try to include both early adopters as well as our skeptical team members. By including those who are not on board yet, or don’t think a change is needed, we can better understand what challenges we face as we try to expand the program. And in this way, hopefully we can engage our skeptics and even help them become believers.”

#### Domain 2: Manage

“Collaborating on the best approach to getting work done” incorporates suggestions such as “listen to the boots on the ground” and to “provide guidance, but allow the team to come up with the solutions.” One panelist noted “We all try to learn from each other and build on our experiences. I try to share successes and failures, my own and the teams, so we all know that these small set-backs should not derail us or the project.” Several panelists also noted the importance of “setting stretch goals” when managing a team, with one panelist recommending the SMART framework (Specific, Measurable, Attainable, Relevant, and Timely) to identify challenging but realistic goals.

#### Domain 3: Sustain

Some panelists noted that it was not only critical to monitor team performance, but to communicate the data. As one panelist commented, “We know that teams and frontline staff are eager to learn the impact of their efforts; providing frequent data to leaders, managers, and frontline staff is essential. What I’ve seen work well is for hospital teams to start their meetings off by reviewing performance data together to develop a collective understanding of how well things are going and whether there are any trends (up or down).” Several panelists also noted the importance of making decisions as a team in order to promote sustainability, with one panelist reflecting, “Individuals in a team are more likely to agree with a shared solution if they feel that their perspectives or ideas were acknowledged and considered.”

#### Domain 4: Train and Feedback

Listening and asking good questions was one of the most highly-referenced behaviors in Domain 4. As one panelist noted, “At our multidisciplinary meetings, I make sure to have everyone participate and be heard. There are usually the vocal team members, and some of the less vocal members can be left unheard due to others that may dominate. I like to spend part of the meeting where we go around the room and each person gets to speak. I also let them know in advance what type of feedback I will be asking from everyone, so that those who are shy or less vocal have time to prepare, and can even submit it in advance so that I can read it to the group if they don’t feel comfortable. Everyone needs a voice!”

#### Domain 5: Manage Team Boundaries

Serving as a liaison between the core project team and other groups and departments was perceived to be an important element of managing team boundaries. A behavior mentioned by several panelists was obtaining senior leadership buy-in through meetings with executives in the early phases of the project, with regular updates about the progress, potential barriers, and successes of the project. Several noted the need for messages to key executives to be “tailored to their particular interests, particularly cost and patient satisfaction” and to “be able to articulate key achievements in less than one minute.”

#### Domain 6: Manage Organizational Context

A key behavior related to managing the organizational context was ensuring access to data the team needs to do their work. To remove barriers to data sources, one panelist commented that his hospital has instituted, “‘Senior Leader Rounding’” where senior leaders visit departments throughout the hospital on a quarterly basis. In this way, “it provides each department [an opportunity] to get to know a different senior leader each quarter, and also an opportunity to ask questions and get information as needed.”

### Round 3: Ranking Leader Behaviors

In Round 3, 24 of 50 leader behaviors achieved group consensus as critical “Must do” behaviors. Table 1 describes the 24 critical behaviors for supporting perioperative improvement teams and examples of how to enact these behaviors. For example, in the design and define domain, high-performing teams *make room for flexibility*, and enact this behavior by ensuring that roles are not too rigidly defined. In the *train and feedback* domain, high-performing teams *appreciate a good failure* and enact this through reflecting on challenges and incorporating that into planning.

**TABLE 1.** Critical Behaviors for Supporting Perioperative Improvement Teams and Examples of How to Enact the Behaviors

Domains	Critical Behaviors	Examples of How to Enact the Behaviors
Domain 1: Design and Define	Develop and articulate a clear team mission	Be explicit about WHERE the desired destination is
	Set explicit objectives	Set clear, specific goals around WHAT to do by WHEN
	Clarify roles	Ensure that team members know WHO is doing WHAT
	Involve key stakeholders and roles	Include as core team members those directly involved and those affected (eg, patients) by the implementation
	Select complementary team members	List desired roles, responsibilities, and skills (e.g., content experts, data experts), and recruit if expertise is missing
	Make room for flexibility	Team members must be prepared to support each other. Roles should not be so rigid that it sacrifices wisdom for compliance
Domain 2: Manage	Collaborate on the best approach to getting work done	Develop team processes that leverage lessons learned about what has worked/ not worked in past team experiences
	Provide project management support	Ensure the team has support of a project manager (other than busy clinicians) to enable success
	Set stretch goals	Set challenging but realistic SMART goals (Specific, Measurable, Attainable, Relevant, and Timely)
	Defer to expertise	Seek opinions from those “in the trenches” doing the work
Domain 3: Sustain	Give them voice	Create a culture of empowerment. Stress that all opinions, positive and negative, will be heard without being judged
	Communicate the data	Begin team meetings by collectively reviewing the data. Report data to frontline staff via managers and department leads
	Lead by example	Do the work to show commitment and motivate others
	Promote team learning	Create open-learning venues; frame it as dynamically learning from successes and failures as opposed to seeking compliance
	Make decisions as a team	Invite ideas from a broad spectrum of stakeholders. Then work together to reach a consensus on the path forward
Domain 4: Train and Feedback	Respect and value each team member	Valuing team members equally is essential and will build confidence and buy-in from the team
	Listen and ask good questions	Pause, listen, then offer thoughts. Ask open-ended questions, inviting input. Give everyone time to speak at meetings
	Create a space for ownership of the work	Have team members present their own work. Develop goals and timelines together as a team, not as a top-down approach
	Have a structured approach to learning	Plan forward, reflect back, and act on the learning. Periodically assess team strengths, weaknesses, opportunities, and threats
	Appreciate a good failure	Implementations often start and stall. When planning next steps, it is vital to learn from what did and did not work
	Respond with encouragement	Be aware of “initiative fatigue.” Make every team member feel their contributions are important
Domain 5: Manage Team Boundaries	Publicize the value of the team’s work	Describe project benefits for other groups in the institution. Report accomplishments at all department/quality meetings
	Obtain senior leadership buy-in	Meet with executives at least quarterly and during budget planning to maintain project support. Regularly email project updates to keep them engaged
Domain 6: Manage Organizational Context	Ensure access to data the team needs to do their work	Engage with executives to remove barriers to data sources (eg, providing resources to participate in data registries, prioritizing changes in the electronic health record system)

## DISCUSSION

Too often perioperative improvement teams fail to achieve their goals.^8^ Frequently this is ascribed to resistance to change or lack of resources.^9,10^ We argue that taking time to preplan and continually reflect on the “how” of enacting team leadership behaviors within each teamwork domain is an important, yet often overlooked, way to engender success. This study integrated recommendations from thought and team leaders entrenched in perioperative work, producing a set of practical behaviors to consider when planning and implementing perioperative improvement initiatives. Based on our findings, leaders should spend time on designing the team, creating a shared vision and collaborative approach to “getting work done,” and having a structured training and feedback process. Although our findings point to areas of consensus across diverse roles, the small number of participants in each role may have obscured differences in role-specific priorities, limiting the specificity of behaviors generated. Nevertheless, by identifying critical team behaviors and distilling practical tips from experts, this work highlights key drivers of team performance in perioperative improvement efforts. As such, this study contributes a framework for translating the science of teams and leadership into actionable guidance.
